# Antioxidant Activity Improvement of Apples Juice Supplemented with Chitosan-Galactose Maillard Reaction Products

**DOI:** 10.3390/molecules24244557

**Published:** 2019-12-12

**Authors:** Jawhar Hafsa, Mohamed ali Smach, Mansour Sobeh, Hatem Majdoub, Aziz Yasri

**Affiliations:** 1 Faculty of Medicine Sousse, Department of Biochemistry, University of Sousse, Sousse 4002, Tunisia; dalifms@live.fr; 2 AgroBiosciences Research Division, Mohamed VI Polytechnic University, lot 660-Hay Moulay Rachid, ben-Guerir 43150, Morocco; Mansour.sobeh@um6p.ma (M.S.); Aziz.yasri@um6p.ma (A.Y.); 3 Laboratory of interfaces and advanced materials, Faculty of Science of Monastir, University of Monastir Monastir 5000, Tunisia; hatemmajdoub.fsm@gmail.com

**Keywords:** chitosan, galactose, Maillard reaction, apple juice, antioxidant

## Abstract

Chitosan-galactose Maillard reaction (CG) were prepared by heating at 100 °C for 3 hrs in a model system containing chitosan (CH) and 1%, 1.5% and 2% (*w*/*v*) of galactose. The results showed that the absorbance at 294 and 420 nm, the fluorescence intensity and the color differences of CG Maillard reaction products (MRPs) increased significantly with the increase of galactose concentration, which indicated the development of MRPs. In addition, FT-IR analysis showed that the degree of deacetylation of CG-MRPs was reduced with the increasing galactose ratio by the schiff base (–C=N) formation, indicating that the galactose has been attached to the amino group of chitosan. Likewise, the antioxidant activities (DPPH, chelating ability and reducing power) of CG-MRPs were investigated. Notably, the effect of galactose concentration in CG-MRPs was found to enhance the antioxidant activity, indicating that CG-2% exhibited the highest antioxidant activity in the range of 0.25–2.0 mg/mL. Furthermore, the apple juice supplemented with CG-MRPs could significantly improve the antioxidant activities, and CG-2% in apple juice showed the better antioxidant capacity at the concentration of 1.0 mg/mL. Thus, we conclude that CG-MRPs addition may greatly improve the antioxidant quality of apple juice.

## 1. Introduction

Chitosan is a natural biopolymer of D-glucosamine and N-acetyl-glucosamine, produced by alkaline deacetylation of chitin and extracted from shellfish and cell walls of some fungi and microorganisms [1,2]. Chitosan has attracted more attention and has been broadly applied in the area of medicine, pharmaceuticals, biotechnology, agriculture and functional food [1,2,3,4]. Currently, chitosan and its derivatives have been recommended as dietary supplements for their confirmed biological properties, low toxicity and biodegradability. Likewise, its antimicrobial and antioxidant properties have attracted more attention as a potential of food preservative of natural products source [4,5,6,7]. Definitely, by chemical modification, chitosan derivatives with specific functions (i.e., improved bioactivity and solubility) can be performed through different chemical reactions, including the acylation reaction [8], reductive alkylation [9] and Maillard reaction [3,7,10,11].

Maillard reaction (MR) is a non-enzymatic and spontaneous browning reaction between amine groups of amino acid and carbonyl groups of reducing sugars [12]. Maillard reaction products (MRPs) are the result of MR, produced widely in food during heating processing, modifying food properties such as flavor, color and stability [13]. The MR has a great potential to become an industrial method to engender antioxidant compounds with several application in food fields [14]. Therefore, due to the presence of amino group, chitosan can participate in MR by reacting with the carbonyl group of reducing sugar. It was also reported that chitosan-MRPs generated by heating with monosaccharides improved solubility and showed significantly higher antimicrobial and antioxidant activity than native chitosan [7,11,14].

Recently, many studies have used chitosan-MRPs for preserving and enhancing nutritional value and the quality of food. For example, Zhu et al. [5] suggested that the chitosan-xylose MRPs could be used as a novel preservative for semi-dried noodles. Moreover, Bakry et al. [15] reported that chitosan-glucose MRPs prepared at 120  °C for 40  min could be a good approach to preserve the freshness of *Ctenopharyngodon idellus* fillets and prolonging shelf-life during cold storage. Finally, a previous study showed that chitosan increased antioxidant activity and inhibits yeasts and molds in apple juice [16,17], and several studies have tried to improve the nutritional value and antioxidant properties of apple juice [18].

In this study, the extent of MR on the physicochemical and antioxidant proprieties of chitosan-galactose MRPs was investigated. In addition, the improvement of an antioxidant effect in apple juice of chitosan galactose MRPs was also tested.

## 2. Results and Discussion

### 2.1. Effect of Galactose Concentration on the Extent of the Maillard Reaction

**Figure 1:**
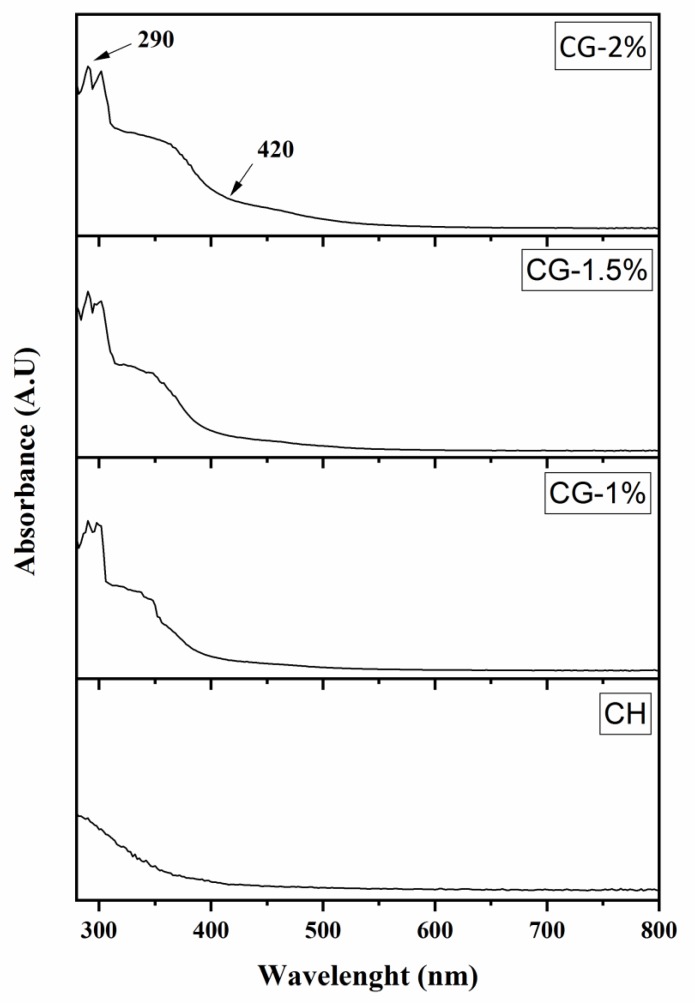
UV–vis spectra (290–800 nm) obtained from chitosan (CH) and chitosan–galactose Maillard reaction (CG-1%, 1.5% and 2%).

**Figure 2:**
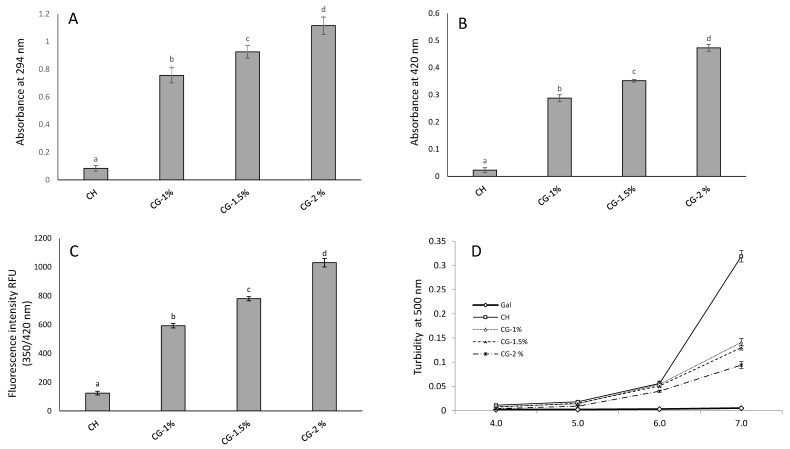
(**A**) Absorbance at 294 nm, (**B**) browning (420 nm), (**C**) fluorescence intensity (350/420 nm) (**D**) and solubility of chitosan (CH) and chitosan–galactose Maillard reaction products (CG-1%, 1.5% and 2%) after heating at 100 °C for 3 h. Values are given as mean ± SD (*n* = 3). Different letters indicate significantly different (*p* < 0.05).

During the development of the MR, colorless intermediate and final brown compounds, which absorb highly UV and visible light are formed [19]. In fact, the extent of the MR can be detected at 294 nm and at 420 nm for the intermediate and the final stages, respectively [11,20]. Furthermore, the fluorescent products can be detected and considered as precursors of brown melanoidins [20]. To explore the effect of MR on CG-MRPs, the UV-vis absorption spectra of chitosan and CG at different concentrations were also investigated. We could conclude that pure chitosan showed no absorption peak in the region 400 nm to 500 nm (Figure 1), while an absorption peak at 420 nm was observed for all CG-MRPs; which can be attributed to the development of brown compounds. The same observations with high absorption peaks were obtained at the UV wavelength, with an optimum Abs at 290 nm associated to the development of colorless intermediate compounds. In addition, two wavelengths at 294 nm and 420 nm were also investigated. The results showed that the absorbance (Abs) at 294 and 420 nm of CH and CG-1%, 1.5% and 2% were 0.083, 0.756, 0.926 and 1.116 and 0.023, 0.288, 0.352 and 0.473, respectively (Figure 2A,B). The values of Abs at 294 nm were higher than the values obtained at 420 nm, suggesting that more intermediate compounds are developed than brown compounds. Similar results were obtained by Kosaraju, Weerakkody and Augustin [21] who reported that the heating of CH at 98 °C with glucose, give higher levels of intermediate products than final stage browning products; in addition, Zhang et al. [11] reported that the Abs at 284 and 420 of 1% CH and 0.5%, 1% and 1.5% of fructose (FS) increased with the increase of reaction time and FS concentration. Hence, we could conclude that the values of CG-MRPs increased significantly (*p* < 0.05) along with increase of Gal concentration.

Fluorescent compounds formed during MR, are not only precursors, but also final products of the reaction [22]. Results showed that the fluorescence intensity (FI) of CG increased significantly (*p* < 0.05) along with the increase of Gal concentration suggesting the formation of MRPs (Figure 2C). The FI of CH and CG-1%, 1.5% and 2% of Gal were 124, 592, 780 and 1029, respectively. Regarding the results obtained, we could conclude, that the rate of reaction increased with the increase of Gal concentration. Additionally, Chung, Kuo and Chen [23] and Gullón et al. [10] reported that the saccharide concentration is an important key factor, and they demonstrated that the double saccharide concentration induces a duplication effects on the Abs at 294 and 420 nm. Considering the results, heated CH with 2% (*w*/*v*) of Gal, was the most efficient concentration to increase the reaction rate.

Chitosan is only soluble in organic medium, which decreases and limits its utility and application in food fields [23]. One of the benefits of MR is to obtain a chitosan is easily miscible with a variety of compounds in aqueous solutions. As shown in Figure 2D, chitosan show a good solubility in acidic medium, which decreases considerably at neutral pH. Nevertheless, the solubility of all CG-MRPs was improved after MR by the diminution of turbidity at 500 nm. Indeed, this can be explained by the reduction of intra- and/or intermolecular hydrogen bonds after the reaction, which leads a good solubility of CG-MRPs [24]. Indeed, substituting hydrogen atoms of amino groups by hydrophilic compound such as galactose, can improve the solubility of CH [23].

### 2.2. Color Measurement of CG-MRPs

Color measurement of CG-MRPs is a useful tool to determine and evaluate the effect of galactose on the extent of MR. The CIELab coordinates and the total difference (L *, a *, b * and ΔE *) of CG-MRPs color are reported in Table 1. Results showed that the heated CH had a slightly yellow green appearance and its L, a and b values were 89.81, −0.24 and 0.10, respectively, but the color was changed when Gal was added with chitosan (Table 1). In fact, all the parameters were affected by the addition of the Gal. In addition, increasing of Gal concentration induced a significant decrease (*p* < 0.05) in lightness and denoted greener (−a *) and more yellow (+b *) color, indicated more browning color at the final stage of MR. The total color difference ΔE* value is an indicator to express the capacity of human eyes to discriminate the color of a sample [11]. Results showed that ΔE * was significantly increased (*p* < 0.05) with increasing of Gal concentration, and the ΔE * of heated CG-1%, 1.5% and 2% were 5.46, 6.09 and 7.59, respectively, indicate that the increases of Gal concentration induced a significant increase in the extent of MR.

### 2.3. FT-IR Analysis of CG-MRPs

**Figure 3:**
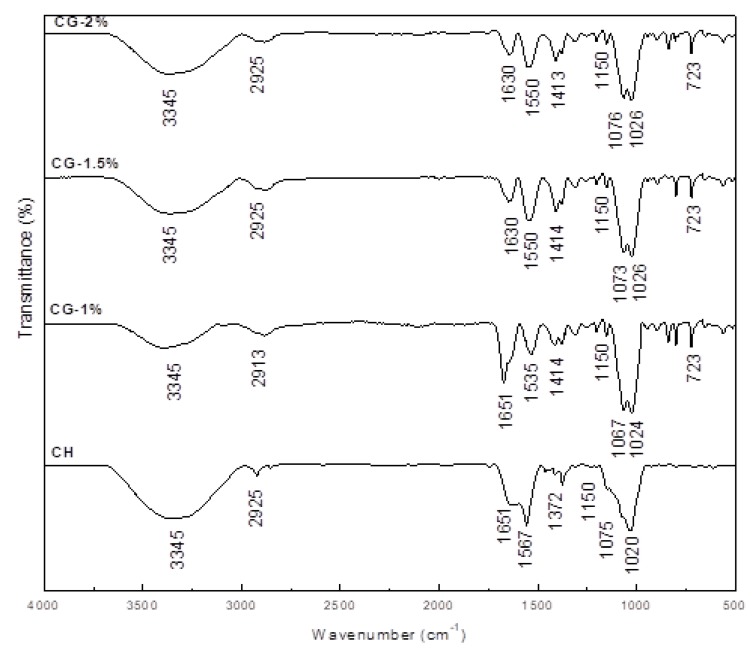
FT-IR spectra (500–4000 cm^−1^) obtained from of chitosan (CH) and chitosan–galactose Maillard reaction products (CG-1%, 1.5% and 2%).

The structural characteristics of CH and CG-MRPs are presented in Figure 3. The FT-IR spectra showed an absorption bands at 3345 cm^−1^ and 2925 cm^−1^ attributed to –NH, –OH and –CH stretching vibration. Peaks at 1651 and 1567 cm^−1^ were assigned to the presence of a carbonyl group and amide II of CH, respectively [2,25].

After MR, we could observe some changes in the absorption bands of CG-MRPs compared to CH, and the absorption peak at 1651 cm^−1^ decreased and shifted to 1630 cm^−1^ suggesting the Schiff base (–C=N) formation between the amino groups of chitosan and the reducing termination of Gal [10]. Peak at the 3345 cm^−1^ became less wider suggesting the interaction between chitosan and carbonyl group of Gal. In addition, the increase in absorption band at 1075 cm^−1^ (–C–O stretching) can be observed after reaction with Gal. Kosaraju et al. [21] and Gullón et al. [10] also could observe these changes using chitosan-glucose MR. Likewise, an increase in the band at 1414 cm^−1^ of CG-MRPs compared to CH produced by the increase of the of –CH_2_ number after the introduction of Gal in the CH molecule. In addition, the profile of all CG-MRPs exhibited more pronounced variations between the bands 500 and 1400 cm^−1^ than native CH. In fact, this region represents the finger print region of the Gal and this variation indicates that modifications were produced [10]. Finally, the DDA (%) of CH and CG-1%, 1.5% and 2% were 79.33%, 65.62%, 56.76% and 48.08%, respectively (Table 1). Additionally, the reducing of CG-MRPs DDA could be explained by the attachment of Gal into chitosan chain.

### 2.4. Antioxidant Activities of CG-MRPs

#### 2.4.1. DPPH Radical-Scavenging Activity

**Figure 4:**
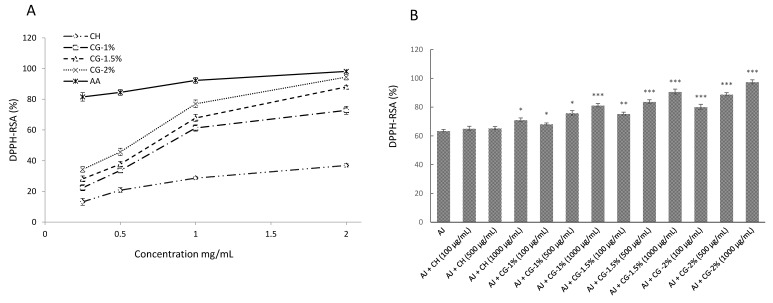
(**A**) DPPH radicals scavenging effects of chitosan (CH), chitosan–galactose Maillard reaction products (CG-1%, 1.5% and 2%) and ascorbic acid (AA). Each value is presented as mean ± SD (*n* = 3). (**B**) DPPH radicals scavenging effects of CH and CG-1%, 1.5% and 2% in apple juice. Each value is presented as mean ± SD (*n* = 3). * *p* < 0.05; ** *p* < 0.01 and *** *p* < 0.001 compared to the control (apple juice).

DPPH-radical-scavenging activity (RSA) offers the first approach for evaluating the antioxidant activity of a compound. It is a simple, inexpensive and rapid widely used method to measure the capacity of compounds to act as hydrogen donors or free radical scavengers [26]. DPPH-RSA of the CH, CG (1%, 1.5% and 2%) and ascorbic acid (AA) was measured at various concentrations as shown in Figure 4A. The AA and CG-2% exhibited the highest RSA, followed by CG-1.5%, CG-1% and CH; and their DPPH-RSA were 98.1%, 94.4%, 88.0%, 72.8% and 36.9% at the concentration of 2.0 mg/mL, respectively. Notably, the effect of Gal concentration was found to influence the DPPH-RSA of CG-MRPs, indicating that CG-2% exhibited the highest scavenging activity than other CG-MRPs and CH in the range 0.25–2.0 mg/mL. Similar results were observed by Zhang et al. [11] who, reported that the DPPH-RSA of CH-FS (0.5%, 1% and 1.5%) increased with the increase of reaction time and FS concentration. In fact, although the number of reactive amino group of CG decreased due to the MR, the radical-scavenging activity of CG-MRPs was higher than that of CH. Hence, the enhanced ability of CG-MRPs on the DPPH radical scavenging may be due to the attachment of N-alkylation and Schiff-base on CH [27].

Figure 4B showed the scavenging potential of CG-MRPs in apple juice. Indeed, apple juice (control) reduced by 63.56% of the DPPH radicals, while CG-MRPs added in apple juice exhibited significant (*p* < 0.05 to 0.001) inhibitory effects toward DPPH radical compared to the control except the CH at 0.1 and 0.5 mg/mL. Overall, we were able to conclude that CG-2% in apple juice had higher RSA, and this was attributable to the stronger hydrogen-donating ability (HDA) of CG-MRPS in apple juice, at the concentration of 0.1 to 1.0 mg/mL. Furthermore, the CG-2% in apple juice exhibited the highest scavenging activity toward DPPH radicals (97.9%) at a concentration of 1.0 mg/mL. The data herein on the DPPH-RSA of CG-MRPs in apple juice revealed that the intermediate and brown compounds produced after MR contributed significantly toward the observed antioxidant effect.

#### 2.4.2. Chelating Effects on Ferrous Ions

**Figure 5:**
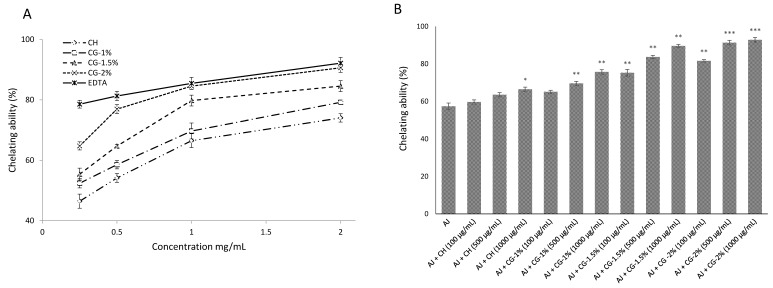
(**A**) Chelating effects on ferrous ions of chitosan (CH), chitosan–galactose Maillard reaction products (CG-1%, 1.5% and 2%) and EDTA. Each value is presented as mean ± SD (*n* = 3). (**B**) Chelating effects on ferrous ions of CH and CG-1%, 1.5% and 2% in apple juice. Each value is presented as mean ± SD (*n* = 3). * *p* < 0.05; ** *p* < 0.01 and *** *p* < 0.001 compared to the control (apple juice).

The generation of reactive oxygen species can be catalyzed by transition metal ions, and results lipid peroxidation damage. Therefore, it should be mentioned that one important mechanism of antioxidant activity is to inhibit the generation of free radicals by chelating ions instead of directly scavenging them, and especially ferrous ion, which is the most active pro-oxidant in the food system [28]. The Fe^2+^ chelating ability of CG-MRPs was measured by the iron–ferrozine complex formation. As presented in Figure 5B. At a concentration of 2.0 mg/mL, results showed that the ferrous metal ions chelating ability of CG-2% (90.6%) was almost equal to the EDTA (92.1%), but better than that of CG-1.5%, CG-1% and CH, which exhibited 84.5%, 79.1% and 74.0% respectively, at the same concentration. Results showed that all the CG-MRPs had an effective capacity for iron binding.

Figure 5B reveals that the ferrous ion-chelating ability of samples in apple juice was significantly higher and correlated with the Gal concentration after MR, except for CH at the concentration 0.1 and 0.5 mg/mL and CG-1% at the concentration 0.1 mg/mL. Results showed that the chelating effect of apple juice was 57.3%, and CG-2% exhibited the highest chelating activity of 92.9%, at the concentration of 1.0 mg/mL. In fact, the most active pro-oxidants present in food systems are ferrous ions and the strong chelating ability of CG-MRPs would be beneficial to improve the food quality [16,28].

#### 2.4.3. Reducing Power Assay

**Figure 6:**
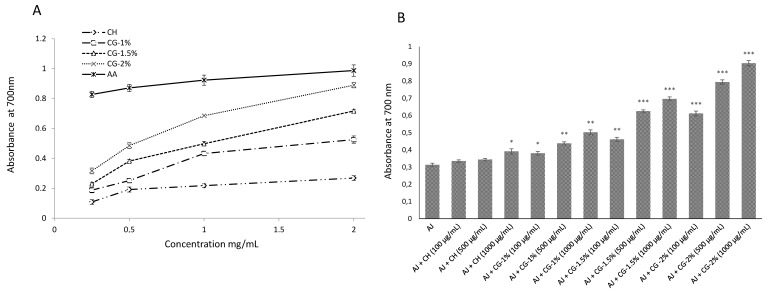
(**A**) Reducing power of chitosan (CH), chitosan–galactose Maillard reaction products (CG-1%, 1.5% and 2%) and ascorbic acid (AA). Each value is presented as mean ± SD (*n* = 3). (**B**) Reducing power of CH and CG-1%, 1.5% and 2% in apple juice. Each value is presented as mean ± SD (*n* = 3). * *p* < 0.05; ** *p* < 0.01 and *** *p* < 0.001 compared to the control (apple juice).

The reducing power is associated with the presence of hydrogen or electron-donating groups, which react with free radicals in order to stabilize and trap radicals [11]. Reducing power of antioxidant or food components are highly associated with their electron donating capacity in the antioxidative system [29]. As shown in Figure 6A, reducing power increased in a dose-dependent manner, and the highest Abs rate of CH, CG (1%, 1.5% and 2%) and AA was 0.268, 0.526, 0.716, 0.889 and 0.987, respectively at the concentration of 2.0 mg/mL (Figure 6A). In fact, the reducing power of CG-MRPs increased with the increasing of the galactose concentration. This can be explained by the HDA of CH that trap free radical chain and donates a hydrogen atom [30]. In addition, after MR, intramolecular hydrogen bonds of CH were broken with more exposed hydroxyl groups that exhibited enhanced and strong HDA. Finally, the intermediate stages products are known as reductones and exhibit high reducing power due to their good HDA.

Figure 6B showed the Abs at 700 nm of CG-MRPs in apple juice. The reducing power of the apple juice was 0.313, while all of CG-MRPs in apple juice exhibited significant Abs rate at 700 nm compared to the control except the CH at 0.1 and 0.5 mg/mL. The CG-2% in apple juice has higher Abs than the other CG-MRPs, which fact is attributable to its HDA and high Abs at 294 nm (intermediate compounds). In fact, the CG-2% in apple juice exhibited the best reducing power (0.904) at a concentration of 1.0 mg/mL. The improved reducing power of CG-MRPs could be explained by the better RSA and the contribution of the advanced MRPs [31].

## 3. Materials and Methods

### 3.1. Preparation of the Chitosan–Galactose Maillard Reaction

The preparation of chitosan–galactose (CG) by the MR was realized according to the method of Gullón et al. [10]. The solution was prepared by dissolving 1% (*w*/*v*) of chitosan in 1% (*v*/*v*) acetic acid, and the pH was adjusted to 5.5 by adding 0.1 N NaOH. Therefore, 0%, 1%, 1.5% or 2% of galactose (*w*/*v*) were added under stirring to each chitosan solution. The mixtures were put inside a test tubes for heating at 100 °C for 3 h in water bath. Reactions were stopped by keeping the solution in an ice bath, and the CG-MRPs were obtained after dialysis against deionized water using dialysis membrane with molecular weight cut-off of 12–14 kDa, and CG-MRPs solutions were freeze-dried.

### 3.2. Extent of the Maillard Reaction

The CG-MRPs were collected and submitted for spectrophotometric analysis at 294 and 490 nm using UV/vis spectrophotometer (Jenway Model 6305, Barloworld Scientific, Dunmow, UK), according to Ajandouz et al. [19]. Therefore, the fluorescence intensity of samples was measured at an excitation wavelength of 350 nm and an emission wavelength of 420 nm using a spectrofluorometer (Versa Fluor spectrofluorometer, Bio Rad), according to Morales and Jimenez-Perez. [32]. UV-vis absorption spectra from 800 to 290 nm were carried out using a UV-vis recording spectrophotometer (Perkin-Elmer Lambda 1050). Distilled water was used as a reference.

Color of MRPs was measured using a colorimeter (Spectraflash 600 plus, Data-color International, Lawrenceville, NJ, USA). The CIE (Commission Internationale de l’Eclairage) values recorded were: L* = lightness (0 = black, 100 = white); a*(−a* = greenness, +a* = redness) and b*(−b* = blueness, +b* = yellowness). Color differences (ΔE) were calculated by the following Equation (1):

(1) $$\Delta E=\sqrt{\Delta a\;*\;2+\Delta b\;*\;2\;+\Delta L\;*\;2}$$

Measurement of solubility of CH and CG-MRP (0.1%) in 0.2 M phosphate buffer at various pH values of 4.0, 5.0, 6.0 and 7.0, was investigated. Solubility was estimated by measuring the turbidity of the solutions at 500 nm [33].

### 3.3. FT-IR Analysis

The structural characteristics of CG-MRPs were determined by Perkin Elmer Spectrum Two ATR-FTIR at the absorbance mode from 4000 to 500 cm^−1^. The degree of deacetylation (DDA%) of different samples was calculated using baseline method as proposed in literature [34]. 

### 3.4. Antioxidant Activities

#### 3.4.1. 2,2-Diphenyl-1-Picrylhydrazyl (DPPH) Radical-Scavenging Activity

DPPH radical-scavenging activity of CG-MRPs was achieved regarding to the method of Huang et al. [26]. One mL of methanolic DPPH solution (0.2 mM) was mixed with 1 mL of CG-MRPs in 0.5 % (*v*/*v*) acetic acid, vortexed and left for 30 min. The absorbance was measured at 517 nm using the same UV/vis spectrophotometer. The percentage of DPPH radical-scavenging activity (DPPH-RSA) was calculated as the following Equation (2):

(2) $$\mathrm{DPPH}-\mathrm{RSA}\;(\%)\;=\;(1\;-\;\frac{{Abs}_{\mathrm{sample}}}{{Abs}_{\mathrm{control}}})\;\times \;100.$$

#### 3.4.2. Chelating Ability on Ferrous Ions

Chelating ability of samples was determined by the method of Dinis, Madeira and Almeida. [35]. Firstly, 7 mL of methanol were mixed with 0.1 mL of ferrous chloride (2 mM) and 1 mL CG-MRPs in 0.5% (*v/v*) acetic acid. Thereafter, the reaction was initiated by the addition of 0.2 mL of ferrozine (5 mM). After 10 min, the absorbance was determined at 562 nm against a blank. EDTA was used for comparison. The percentage of inhibition was calculated using the following Equation (3):

(3) $$\mathrm{Metal}\;\mathrm{chelating}\;\mathrm{effect}\;(\%)\;=\;(1\;-\;\frac{{Abs}_{\mathrm{sample}}}{{Abs}_{\mathrm{control}}})\;\times \;100.$$

#### 3.4.3. Reducing Power

Reducing power was determined by the method of Zhang et al. [11]. One mL of CG-MRPs in 0.5% (*v/v*) acetic acid was mixed with 2.5 mL of phosphate buffer (200 mM, pH 6.6) and 2.5 mL of 1% potassium ferricyanide. Then, the reaction mixture was incubated for 20 min at 50 °C, followed by the addition of 2.5 mL of 10% trichloroacetic acid (TCA), and then centrifuged at 840× *g*/min for 10 min. Afterwards, 5 mL of upper layer was mixed with 5 mL of distilled water and 1 mL of 0.1% ferric chloride. The absorbance of the resultant solution was measured at 700 nm and ascorbic acid (AA) was used for comparison.

### 3.5. Antioxidant Activity of Apple Juice Containing MRPs

The antioxidant activity of apple juice was determined according to the method described by Chien et al. [16]. Clear, UHT-treated apple juice, that contained no added preservatives, was purchased from a local retailer. To 45 mL of this apple juice was added 5 mL of CG-MRPs solution at the concentration of 0.1 to 1 g/L, and 5 mL of water to the control. Afterward, apple juices with added samples were mixed vigorously and filtered, and the antioxidant capacities were measured. 

### 3.6. Statistical Analysis

The statistical analysis was performed using SPSS Statistics Software (SPSS for Windows software release 18.0, Chicago, IL, USA). Dunnett’s post hoc tests were used to detect differences among mean values.

## 4. Conclusions

Recently, the Maillard reaction became an effective method to generate efficacious antioxidant compounds, which can be used as food preservative. It can be concluded that chitosan–galactose Maillard reaction products produced in this study were endowed with better antioxidant activities in vitro and in apple juice than chitosan alone. In fact, heated chitosan with 2% (*w*/*v*) of galactose, was the most efficiency concentration to increase the Maillard reaction and the antioxidant activities in vitro and in apple juice. Due to its enhanced properties, the chitosan–galactose Maillard reaction could be considered as promising for its future use as additives in the food industry. However, the antimicrobial properties of chitosan–galactose MRPs in apple juice must be also investigated.

### Highlights

- The chitosan–galactose Maillard reaction products (CG-MRPs) were prepared.
- The extent of CG-MRPs increase along with increases of galactose concentration.
- All CG-MRPs showed better antioxidant activities than chitosan.
- CG-MRPs supplemented in apple juice improved significantly the antioxidant activity.
- The CG-MRPs might be useful for application as a food preservative.

## Figures and Tables

**Table 1:** Degree of deacetylation and color measurement of chitosan and chitosan–galactose Maillard reaction products.

Treatment Group ^a^	DDA (%)	L*	a*	b*	ΔE
**CH**	79.3	89.81 ± 0.17 ^a^	−0.24 ± 0.05 ^a^	0.10 ± 0.04 ^a^	-
**CG-1%**	65.6	85.36 ± 0.14 ^b^	−0.35 ± 0.11 ^b^	0.75 ± 0.05 ^b^	5.46 ± 0.21 ^a^
**CG-1.5%**	56.7	85.04 ± 0.07 ^c^	−0.36 ± 0.10 ^b^	0.92 ± 0.04 ^c^	6.09 ± 0.10 ^b^
**CG-2%**	48.0	83.95 ± 0.07 ^d^	−0.38 ± 0.01 ^c^	1.11 ± 0.06 ^d^	7.59 ± 0.07 ^c^

CH: chitosan; CG-MRPs: chitosan galactose Maillard reaction products. ^a^ In all CG treatments, the Maillard reaction occurred in 1% chitosan solution with the addition of 1%, 1.5% and 2% galactose at 100 °C for 3 h. In the same column, values with the same superscript letter (^a–d^) were not significantly different (*p* > 0.05). Data value is presented as mean ± SD (*n* = 3), for color measurement.
